# Machine‐Learning‐Guided Design of Incommensurate Antiferroelectrics via Field‐Driven Phase Engineering

**DOI:** 10.1002/advs.202523873

**Published:** 2026-02-09

**Authors:** Ke Xu, Xiaoming Shi, Zhaochen Xi, Shouzhe Dong, Changqing Guo, Rongzhen Gao, Letao Yang, Jing Wang, Di Zhou, Houbing Huang

**Affiliations:** ^1^ School of Materials Science and Engineering & School of Interdisciplinary Science Beijing Institute of Technology Beijing China; ^2^ School of Mathematics and Physics University of Science and Technology Beijing Beijing China; ^3^ School of Electronic Science and Engineering Xi'an Jiaotong University Xi'an China

**Keywords:** antiferroelectrics, energy storage performance, machine learning, multi‐scale regulation, phase‐field simulations

## Abstract

Antiferroelectrics, defined by antiparallel polarization configurations, have emerged as promising dielectric materials for high‐performance energy storage applications. The energy storage density and efficiency of antiferroelectrics are governed by the regulation of electric field‐driven antiferroelectric‐ferroelectric phase transitions (and their reversibility). Herein, we propose a machine learning‐guided phase‐field simulation framework to accelerate the design of energy storage ceramics. Taking PbZrO_3_‐based incommensurate antiferroelectrics as an example, we establish quantitative mappings between energy storage density/efficiency and key features (point defect, antiphase boundary energy, grain size, and strain) using XGBoost. The model exhibits high accuracy for energy storage performance prediction, with R^2^ values of 0.99. SHAP interpretable machine learning analysis further deciphers the correlations between multiple features and energy storage properties, while phase‐field simulations clarify the mechanisms. Guided by this framework, we achieve a high energy storage density of 22.1 J/cm^3^ with 96.1% efficiency. This work provides a theoretical foundation and a generalized approach for regulation of antiferroelectric energy storage properties and mechanisms of phase transition.

## Graphical abstract

The key to enhancing the energy storage performance of antiferroelectrics lies in regulating the phase transition and reverse phase transition. A phase‐field‐machine learning framework is employed to predict the energy storage performance of Pb‐based incommensurate antiferroelectrics with multi‐scale regulation strategy, thereby revealing the dynamic mechanisms of the phase transition process.

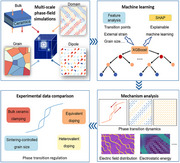

## 1 Introduction

The development of next‐generation dielectric materials with high energy storage density (*W*_rec_) and efficiency (*η*) is an urgent demand in fields such as mobile electronic devices, new energy vehicles, and pulsed power systems [1, 2, 3]. Ferroelectric (FE) ceramics typically exhibit high remanent polarization (*P*_r_), which restricts the improvement of *W*_rec_. Antiferroelectrics (AFEs), characterized by antiparallel polarization configurations resulting in double hysteresis loops and negligible *P*_r_ in the absence of an external electric field, are regarded as key candidates to break through the energy density bottleneck of dielectric materials. Based on the polarization‐electric field (*P*–*E*) loop, *W*_rec_ and *η* during the charge–discharge process can be calculated. The energy storage performance of AFEs is mainly determined by the breakdown electric field (*E*_b_) and nonlinear polarization response. Numerous studies have reported the enhancement of *E*_b_ in AFE ceramics through elements doping [4, 5, 6], multilayer ceramic fabrication [7, 8, 9], and grain engineering [10, 11, 12]. Correspondingly, the key to improving the energy storage performance via polarization response lies in regulating the phase transition points of AFE‐FE (*TP*_AF_) and FE‐AFE (*TP*_FA_) under external electric field [13, 14, 15]. Researchers have increasingly adopted regulatory strategies over the past few years, (e.g., external strain, sintering optimization, and element doping) to regulate the *P*–*E* loops of AFE ceramic bulks [16, 17, 18]. Applying compressive prestress via mechanical clamping increases the reverse phase transition field (*E*_FA_) and reduces hysteresis, yielding significant *W*_rec_ improvement [19]. Low‐temperature sintering‐induced uniform fine grains narrows the gap between phase transition electric field (*E*_AF_) and *E*_FA_, boosting *η* [20, 21]. Accompanying grain boundary volume fraction increase lowers saturated polarization (*P*_s_) and restricts *W*_rec_ enhancement [22]. Element doping effectively modulates AFE polarization behavior: in PbZrO_3_‐based incommensurate AFE systems, A‐site La doping combined with B‐site Sn/Hf doping (medium‐entropy design) achieves 14.8 J/cm^3^ of *W*_rec_ and 90.2% of *η* [23], while La/Sn co‐doping‐induced incommensurate‐commensurate polymorphic AFE domains enable a high *W*_rec_ of 23.7 J/cm^3^ and *η* of 88% [24].

Exploring the microscopic mechanism of AFE polarization regulation still faces challenges. Different from the in‐situ characterization of FE domain switching, the research on AFE‐FE phase transition dynamics needs to balance high external electric field and atomic‐scale resolution. Characterization methods such as transmission electron microscope and piezoelectric force microscope cannot in‐situ capture the antiparallel polarization switching process [25, 26, 27, 28, 29]. In addition, the modification of AFE ceramics involves multi‐scale and multi‐factor cross‐regulation. It is essential to leverage large‐scale datasets to quantitatively establish the mapping correlation between multi‐scale parameters and energy storage performance, while exploring optimal regulatory strategies. Machine learning has demonstrated great potential in predicting the energy storage performance of FEs [30]. Random forest model has been employed to predict and screen the compositions of high‐entropy ceramics [31, 32]. Combined with feature analysis, XGBoost model has achieved high‐accuracy prediction with *P*_r_ of (K_0.5_Na_0.5_)NbO_3_‐based FE ceramics [33]. Additionally, the phase‐field‐CatBoost model has been used to investigate the influence of polar nanoregions on the energy storage performance of BiFeO_3_‐based dielectrics [34]. However, research on the phase transition mechanism of AFEs at the dipole‐scale resolution and the multi‐scale energy storage performance design strategies is still insufficient, which limits the advancement of theoretically guided experimental design.

**FIGURE 1:**
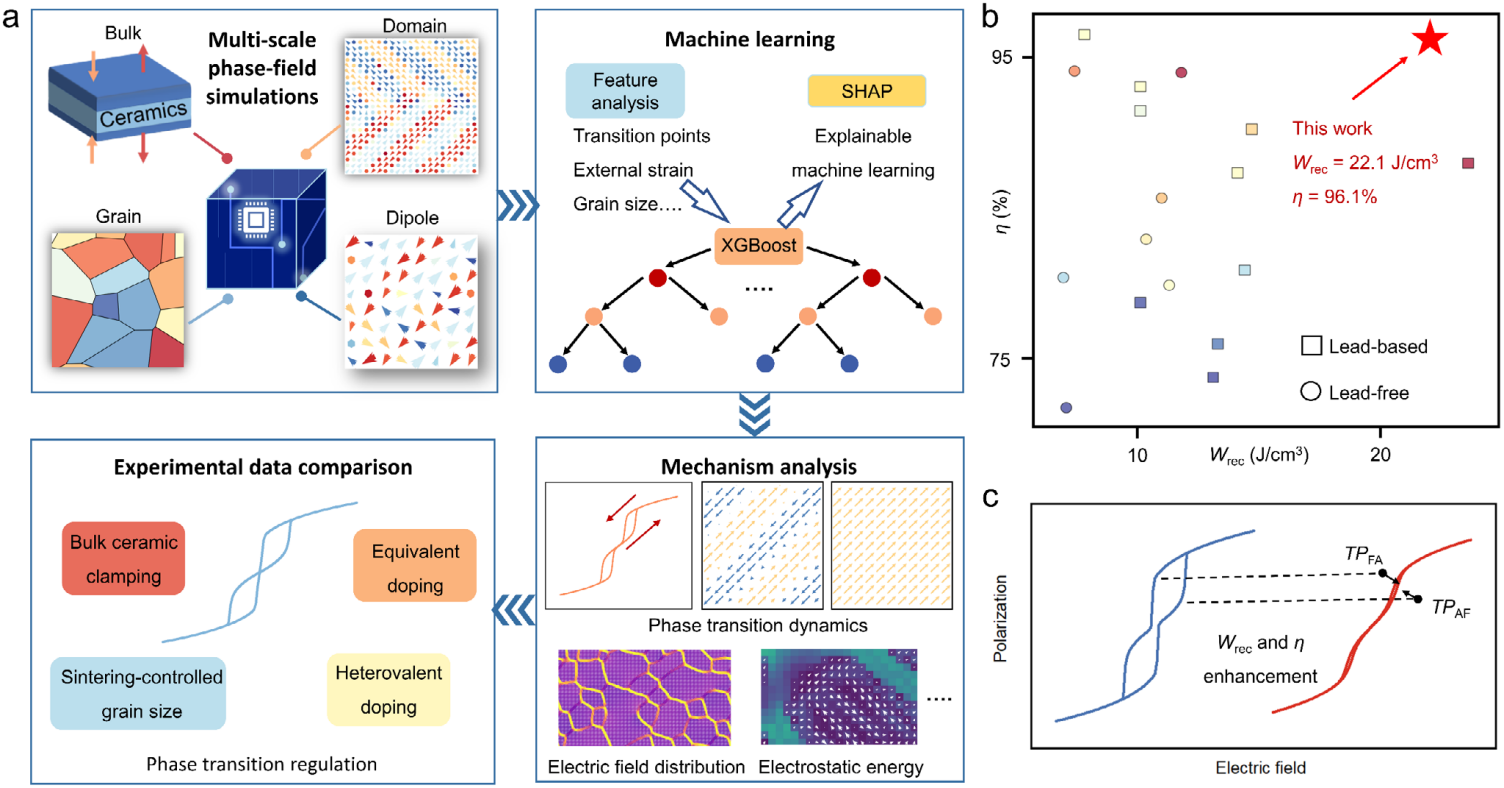
Workflow of multi‐scale regulated high energy‐storage AFE ceramics through phase‐field‐machine learning model. (a) Workflow of phase‐field simulations, machine learning, mechanism analysis and experimental data comparison. (b) Comparison of *W*_rec_ and *η* for antiferroelectric bulk ceramics [4, 8, 10, 24, 41, 42, 43, 44, 45, 46, 47, 48, 49, 50, 51, 52, 53]. (c) Schematic of *P*–*E* loops with improved energy storage performance via phase transition points regulation.

In this work, we adopt a phase‐field‐machine learning coupling framework to reveal the microscopic dynamic mechanism corresponding to the regulation of *TP*_AF_, *TP*_FA_ and construct the mapping correlation between multi‐scale regulation and energy storage performance. The workflow has been shown in Figure 1a. We select PbZrO_3_‐based incommensurate AFEs as an example and introduce five multi‐scale regulatory variables, including point defect concentration (*c*), local inhomogeneous strain (*ε* ^rand^), gradient energy coefficient (*g*), average grain size (*D*), and external strain (*ε* ^ext^) as important features. A high‐throughput dataset of 1280 phase‐field simulation groups has been obtained through independent variable regulation. However, the influence of the cross‐interaction of multiple variables on the *P*–*E* loop and energy storage performance is extremely complex. To further reveal the correlation between the multi‐scale regulation strategy, *TP*_AF_, *TP*_FA_, and energy storage performance, we first conduct Pearson correlation analysis to obtain the basic linear correlations between independent features and energy storage performance‐related parameters. Subsequently, we have constructed an interpretable ensemble decision tree model based on the XGBoost‐Shapley additive explanations (SHAP) framework. This model can not only achieve accurate prediction of *W*_rec_ and *η*, but also analyze the effect of multi‐scale regulation parameters on phase transition points and energy storage performance. Based on the phase‐field‐machine learning model, a high *W*_rec_ of 22.1 J/cm^3^ and *η* of 96.1% (Figure 1b) are achieved with biaxial compressive strain (*ε* ^ext^ = ‐1.0%), large‐sized grain (*D* = 55 nm), large gradient energy coefficient (*g* = ‐0.44 × 10^−10^ J·m^3^·C^−2^), and high point defect concentration (*c* = 3.0). Finally, we have compared the *P*–*E* loops with experimental data and observed consistent trends, which confirms the effectiveness of the proposed research workflow. This study reveals the multi‐scale regulation mechanism of AFE phase transition dynamics and energy storage performance modification, providing a theoretical basis for experimental design.

## 2 Results and Discussion

### 2.1 Phase‐Field‐Machine Learning‐Driven Antiferroelectrics Design

The *P*–*E* loops of AFEs differ from those of FEs, characterized by a double‐hysteresis loop. Therefore, the key to regulating polarization behavior and improving energy storage performance lies in adjusting the coordinates of the *TP*_AF_ and *TP*_FA_ in the *P*–*E* loop. It is generally considered that the convergence of these two transition points toward the middle position is more conducive to achieving high *W*_rec_ and *η*, as shown in Figure 1c. This phenomenon can be explained by the AFE‐FE triple‐well Landau potential theory. The AFE phase exhibits no macroscopic polarization and maintains a low‐energy state without the external electric field. When an external electric field is applied, it can drive the FE phase with lower energy, as indicated by the blue curve in Figure S1. However, the AFE phase still needs to overcome a relatively high energy barrier to complete the phase transition. The key to obtaining AFE ceramics with higher energy storage performance is to reduce the phase transition energy barrier, which facilitates both the AFE‐FE phase transition and the FE‐AFE reverse phase transition, as shown by the red curve in Figure S1.

**FIGURE 2:**
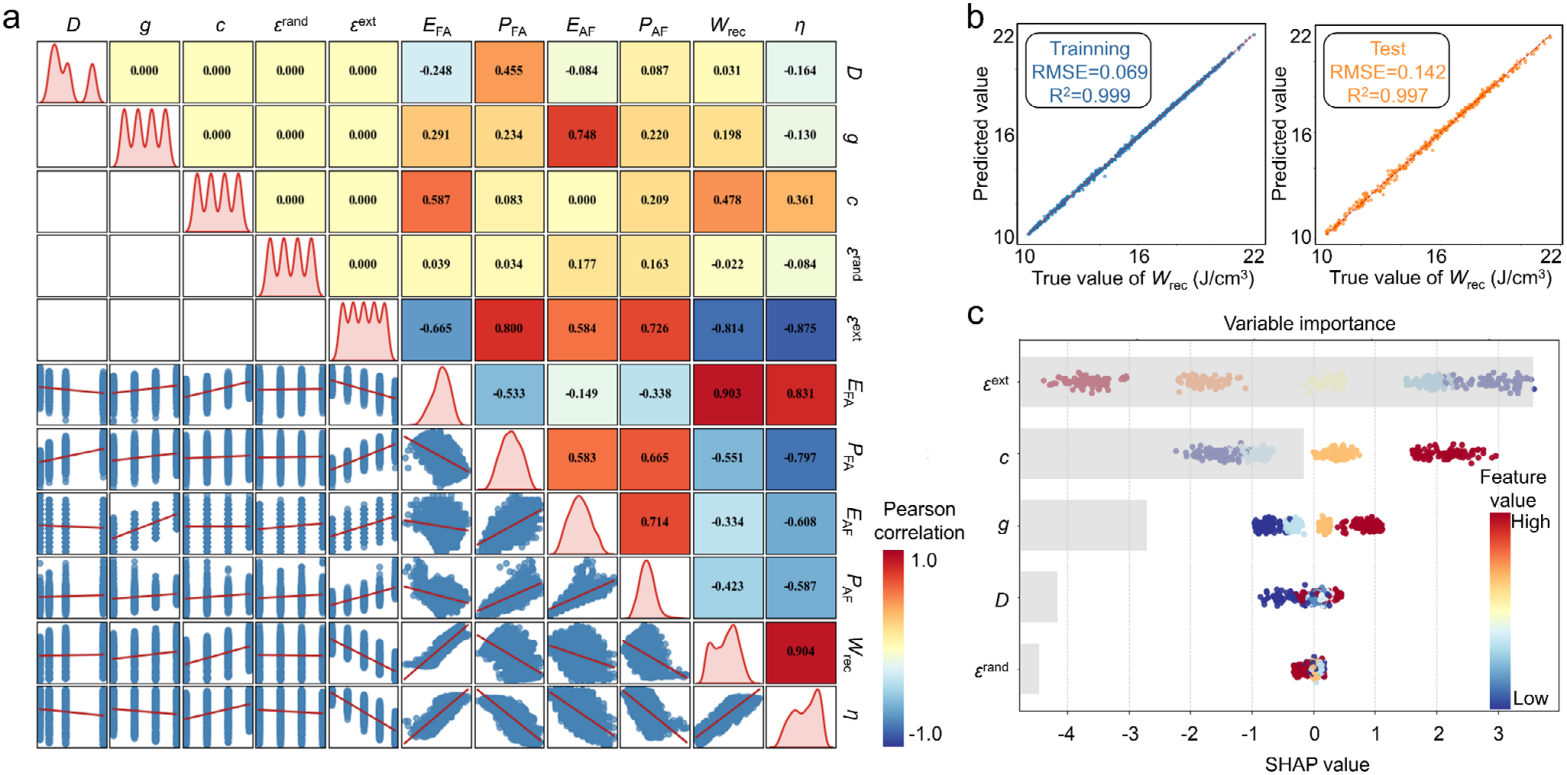
Pearson correlation analysis and XGBoost regression model for decoupling the key factors determining energy storage performance. (a) Pearson correlation matrix, the diagonal shows the kernel density estimation plots for individual variables. The lower triangle presents the linear regression analysis between two different variables (blue scatter points represent the original data, the red line denotes the least squares linear regression fitting line). The upper triangle displays the Pearson correlation coefficients between variables. (b) Comparison of *W*_rec_ between the phase‐field simulations and XGBoost prediction model. (c) SHAP summary plot for the XGBoost model of *W*_rec_ including variable importance and SHAP value.

In the Pearson correlation matrix (Figure 2a), it can be found that the phase‐field simulation input variable with the strongest positive correlation with *W*_rec_ and *η* is point defect concentration (0.478 and 0.361), while the independent variable with the strongest negative correlation is external strain (−0.814 and −0.875). This implies that the improvement of energy storage performance generally follows the rule of increasing point defect concentration and external compressive strain. In addition, the phase transition characteristic variable with the strongest positive correlation with *W*_rec_ and *η* is *E*_FA_, indicating that the increase in *E*_FA_ can significantly enhance energy storage performance. Based on this, 1280 sets of phase‐field simulation data have been input into XGBoost for training the prediction model, with 80% of the data used for the training set and 20% for the test set. As shown in Figure 2b, in the prediction of *W*_rec_, the root mean square error (RMSE) of the training set is 0.069, and the coefficient of determination (R^2^) is 0.999. For the test set, RMSE equals to 0.142 and R^2^ equals to 0.997, indicating high accuracy of the model. In addition, the prediction model for *η* also shows high accuracy, with R^2^ equals to 0.999 for the training set and R^2^ equals to 0.998 for the test set, as shown in Figure S2a. We also perform weight analysis on the key independent variables determining energy storage performance through SHAP analysis. The results in Figure 2c show that external strain is the most important parameter, indicating that it plays a dominant role in regulating the *TP*_AF_ and *TP*_FA_. Furthermore, point defect concentration, gradient energy coefficient, and average grain size are all positively correlated with *W*_rec_, and the local inhomogeneous strain field has priority in influencing *W*_rec_. The influence weights of the above independent variables on energy storage efficiency in descending order are *ε* ^ext^, *c*, *D*, *g*, and *ε* ^rand^ (Figure S2b). Except for *c*, which is positively correlated with *η*, all other variables are negatively correlated with *η*.

We have also compared the prediction accuracy and training efficiency of XGBoost with other ensemble decision tree models such as LightGBM and CatBoost for energy storage performance based on the phase‐field simulation dataset. All three representative ensemble decision tree models have demonstrated good prediction accuracy for energy storage performance, as shown in Figure S3a,b. However, XGBoost has a certain advantage in terms of model training efficiency (Figure S3c). The specific parameter settings for the machine learning model can be found in the Methods section.

### 2.2 Local Field Effect in Phase Transition

**FIGURE 3:**
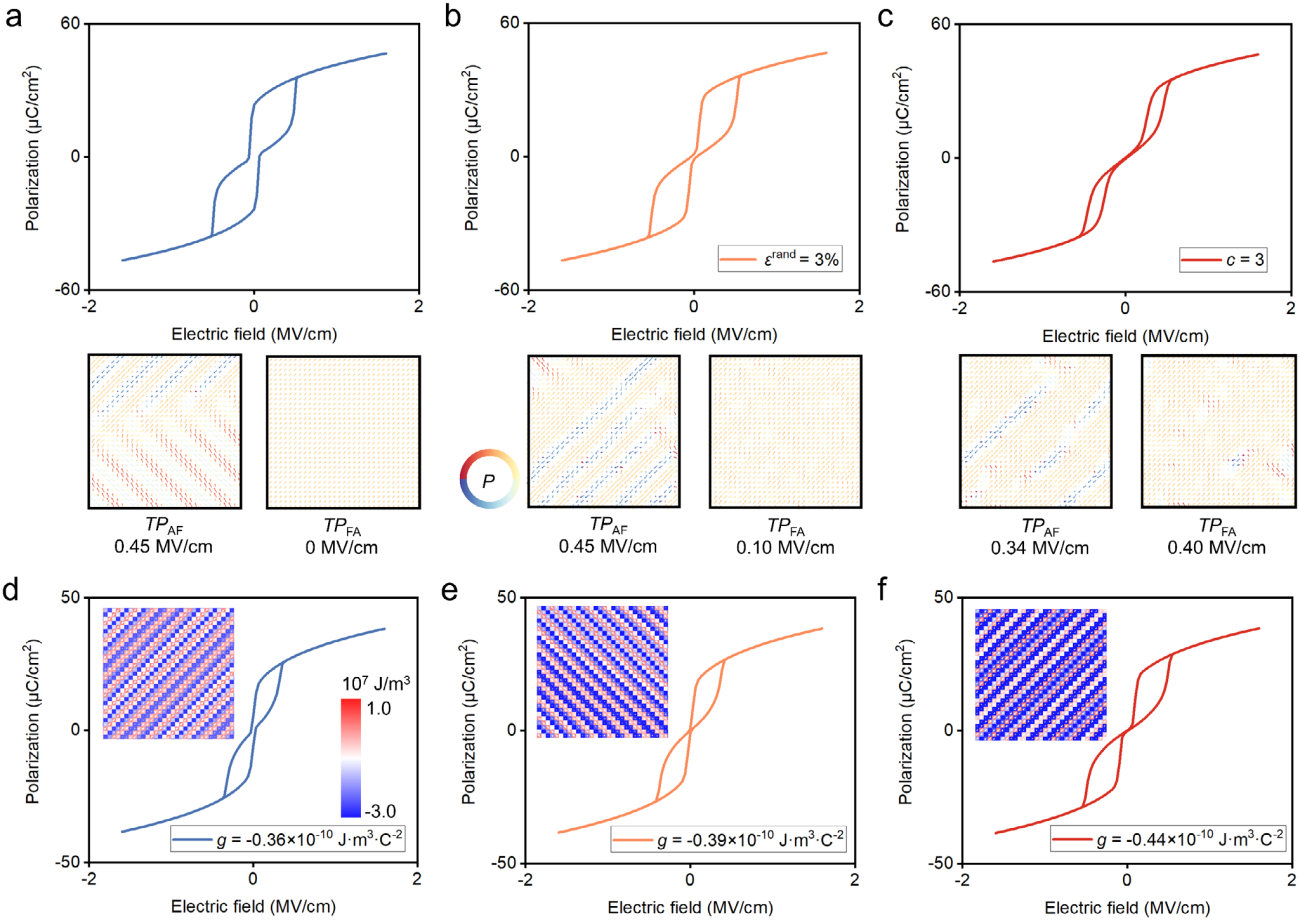
Analysis of the polarization behaviors and domain structure mechanism of antiferroelectric phase transition under dipole‐scale factors. (a–c) Phase‐field simulation on effects of inhomogeneous strain (*ε* ^rand^ = 3%) and point defect concentration (*c* = 3) on *P*–*E* loops and local domain structure of phase transition points. d‐f *P*–*E* loops and gradient energy density distribution of antiphase domain boundary effect.

To reveal the local field effect induced by component design or experimental processes at the dipole scale, we have focused on the influences of local inhomogeneous strain and point defect factors on the regulation of phase transition process in *P*–*E* loops and domain switching (Figure 3a–c). As shown in Figure 3b, when *ε* ^rand^ = 3.0%, the *P*–*E* loop becomes slightly narrower, with a certain improvement in *η*, but the enhancement of *W*_rec_ is not significant. With the increase in point defect concentration (*c* = 3.0), the *P*–*E* loop narrows remarkably, accompanied by a decrease in the *E*_AF_ and an increase in the *E*_FA_ (Figure 3c), leading to significant improvements in both *W*_rec_ and *η*.

To clarify the regulation mechanism at the dipole scale, we simulate the local domain evolution during the phase transition process. As for the domain without local field effect, when *E* ^ext^ increases 0.45 MV/cm, the FE phase nucleates at the AFE domain boundaries and diffuses into the AFE phase, which corresponds to the *TP*_AF_. When the external electric field is removed, the reverse phase transition from FE phase to AFE phase is completed rapidly, resulting in an AFE *P*–*E* loop. The introduction of local inhomogeneous strain field causes the dipoles to deviate from their equilibrium positions, presenting a disordered state. The *E*_FA_ increases to 0.1 MV/cm. Part of the dipole has deviated from the direction of the external electric field, promoting the nucleation of AFE phase. The local electric field generated by point defects induces disordered dipoles at the antiphase domain boundaries (Figure 3c). Although local disordered dipoles reduce AFE anisotropy and phase transition energy barrier, they still exhibit a strong pinning effect at the*E* ^ext^ = 0.4 MV/cm. When the external electric field decreases to near the *TP*_FA_, the AFE domains achieve nucleation at the disordered regions formed by point defects. In contrast to the simulation without local field effects, both the inhomogeneous strain field and point defects transform the electrostatic energy density distribution from an ordered state to a disordered state (Figure S4a–c). The disordered electrostatic energy distribution modifies the nucleation and propagation of the FE and AFE phases, thereby regulating the *TP*_AF_ and *TP*_FA_ (Figure S4b,c).

### 2.3 Antiphase Domain Boundary Effect in Phase Transition

In the AFE phase‐field model, the nearest‐neighbor interaction of polarization is associated with the tilt angle of oxygen octahedra, which can be represented by the gradient energy density coefficient. As the gradient energy density coefficient *g* decreases, *P*–*E* loop gradually transforms into a typical AFE loop with a linear polarization response region, as shown in Figure 3d–f. The *E*_AF_ increases from 0.192 to 0.384 MV/cm, and the *E*_FA_ rises from 0.055 to 0.192 MV/cm, accompanied by an increase in the (reverse) phase transition polarization (Figure S5). This indicates a gradual enhancement of the antiferroelectricity of the system.

To analyze the influence of the gradient energy coefficient on the domain structure, we have plotted the total gradient energy density distribution (Figure 3d–f). In the incommensurate AFE, the total gradient energy corresponding to polarization is mainly determined by the modulation of nearest‐neighbor interactions and next‐nearest‐neighbor interactions. Without an external electric field, parallel nearest‐neighbor polarization shows a high gradient energy density, while the antiparallel polarization has an extremely low one, indicating high AFE stability. This implies that a higher external electric field is required to break the stable antiphase domain boundaries, significantly increasing the *E*_AF_ and *E*_FA_. In addition, FE domains in the saturated state still maintain high total gradient energy density. As the external electric field decreases, the system also rapidly undergoes a reverse phase transition back to the AFE phase.

### 2.4 Grain Size Effect in Phase Transition

**FIGURE 4:**
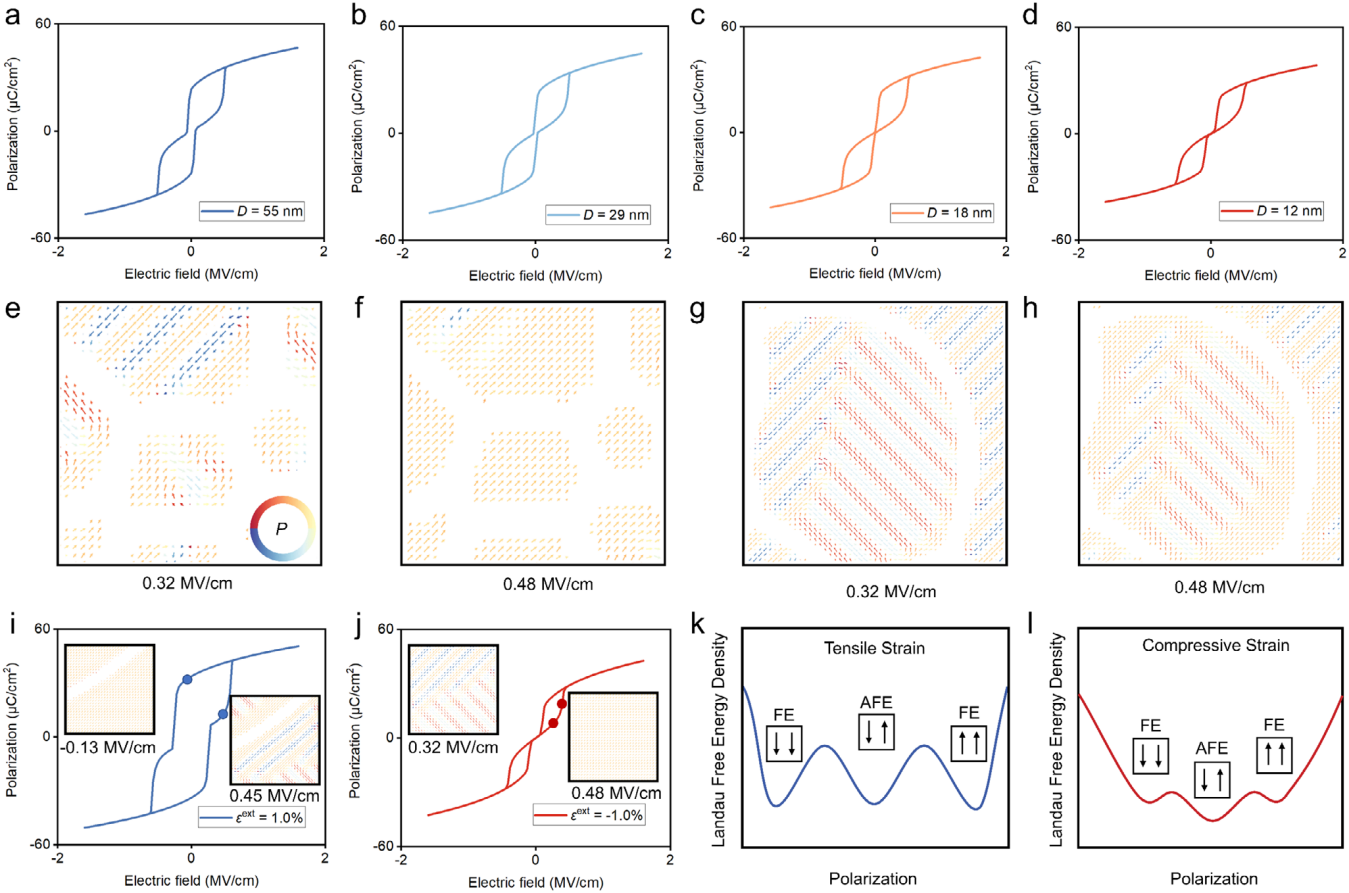
Mechanism analysis of polarization behaviors and domain structure dynamics for energy storage performance regulation by grain effect and external strain. (a–d) *P*–*E* loops with different average grain size. Phase transition dynamics of (e,f) small‐sized grains and (g,h) large‐sized grains. *P*–*E* loops and domain structure evolutions under external electric field with biaxial (i) tensile strain, (j) compressive strain. Schematic of Landau free energy density under biaxial (k) tensile strain, (l) compressive strain.

The grain size effect is also a key factor influencing the AFE phase transition behavior. Here, we construct initial structures of AFE nanocrystals with different average sizes. We fill the grain boundaries with a low‐permittivity layer to approximate the dielectric characteristics of the amorphous phase. Then we investigate the phase transition characteristics of large‐sized and small‐sized grains under the external electric field. As shown in Figure 4a–d, with the average grain size decreasing from 55 to 12 nm, the hysteresis of the AFE *P*–*E* loop decreases significantly, indicating that the reduction in grain size is beneficial to the improvement of *η*. When the external electric field strength increases from 0.32 to 0.48 MV/cm, the small‐sized grains completely transform from the AFE phase to the FE phase (Figure 4e,f). The large‐sized grains exhibit a mixed‐phase structure of AFE and FE without completing the phase transition (Figure 4g,h). Interestingly, we have found that the AFE‐FE phase transition seems to preferentially nucleate near the grain boundaries and gradually diffuse into the interior of large grains. The external electric field shows a significant concentration effect at the grain boundaries with low permittivity (Figure S6a). The electric field strength at the grain boundaries which is vertical to the external electric field direction reaches up to 5.0 MV/cm, while the electric field inside the AFE grains is approximately 1.0 MV/cm, as shown in Figure S6a. The mismatch in interface electric field strength leads to a strong depolarization effect between the interior of AFE phase and the grain boundaries. The AFE stripe domains form flux‐closed domains at the grain boundaries to reduce the local electrostatic energy. The AFE flux‐closed domains lower the phase transition energy barrier, making the FE phase tend to nucleate at the grain boundaries (Figure S6b). The nucleation‐diffusion model can explain why small‐sized grains complete the phase transition earlier than large‐sized grains.

In addition, the reduction in average grain size also leads to a decrease in average polarization. As the average grain size decreases from 55 to 12 nm, the grain boundary volume fraction increases rapidly from 5.5% to 18.5% (Figure S7). The contribution of grain boundaries with low permittivity to the average polarization is almost negligible compared with that of AFE grains. Thus, the *P*_s_ also decreases from which restricts the improvement of *W*_rec_.

### 2.5 External Strain Effect in Phase Transition

The effect of external strain exerts a significant regulatory role on the polarization behavior of AFE ceramics. In bulk ceramics, external strain regulation of uniaxial strain, biaxial strain, and hydrostatic pressure can be achieved through clamping. In multilayer ceramic capacitors, varying *E*_AF_ among multilayer AFEs causes differential strains, creating interlayer tension or compression that adjusts energy storage performance. Herein, we have applied biaxial tensile or compressive strains to the domain structure along the *X*‐axis and *Y*‐axis, while applying an external electric field along the [11]‐direction. When the external tensile strain reaches 1%, the *P*–*E* loop exhibits characteristics close to the FE phase (Figure 4i). To study the corresponding evolution of the domain structure, we simulate the local polarization distribution during the domain switching process, as shown in Figure 4i. When *E* ^ext^ = 0.45 MV/cm, a metastable AFE phase appears near the grain boundaries, and the overall structure exhibits an AFE‐FE mixed‐phase. As the electric field strength increases further, the metastable AFE phase transforms into an FE phase aligned with [11]‐direction. When the electric field is completely removed and then increased in the reverse direction to *E* ^ext^ = ‐0.13 MV/cm, the FE phase remains stable. The mechanism of FE‐like *P*–*E* loops can be attributed to the reduction in the Landau free energy of the FE phase induced by biaxial tensile strain. When the biaxial tensile strain reaches 1%, the Landau free energies of the AFE and FE phases are close, as illustrated in Figure 4k. However, under the external electric field along the [11]‐direction, the Landau free energy of the AFE phase increases while that of the FE phase decreases, thus enabling the stability of FE phase.

In contrast to biaxial tensile strain, when *ε* ^ext^ = −1%, the *P*–*E* loop shows a typical AFE double‐hysteresis, with a significant improvement in *η*. The biaxial compressive strain suppresses the increase in polarization along in‐plane directions. It is difficult for the polarization to rotate toward the external electric field direction, resulting in a noticeable decrease in average polarization and a certain enhancement in the stability of the AFE phase (Figure 4j). When the external electric field exceeds *E*_AF_, the AFE stripe domains quickly overcome the energy barrier and transform into the FE phase. However, the average polarization remains low under the constraint of biaxial compressive strain, as shown in Figure 4l. When the external strain changes from biaxial tension to compression, the *TP*_AF_ and *TP*_FA_ converge toward the middle position overall, which is conducive to the improvement of energy storage performance, as shown in Figure S8. In general, biaxial tensile and compressive strains of up to 1% can significantly alter the *TP*_AF_ and *TP*_FA_, enabling the regulation of energy storage performance.

### 2.6 Comparative Analysis of Phase‐Field Simulations and Experimental Results

**FIGURE 5:**
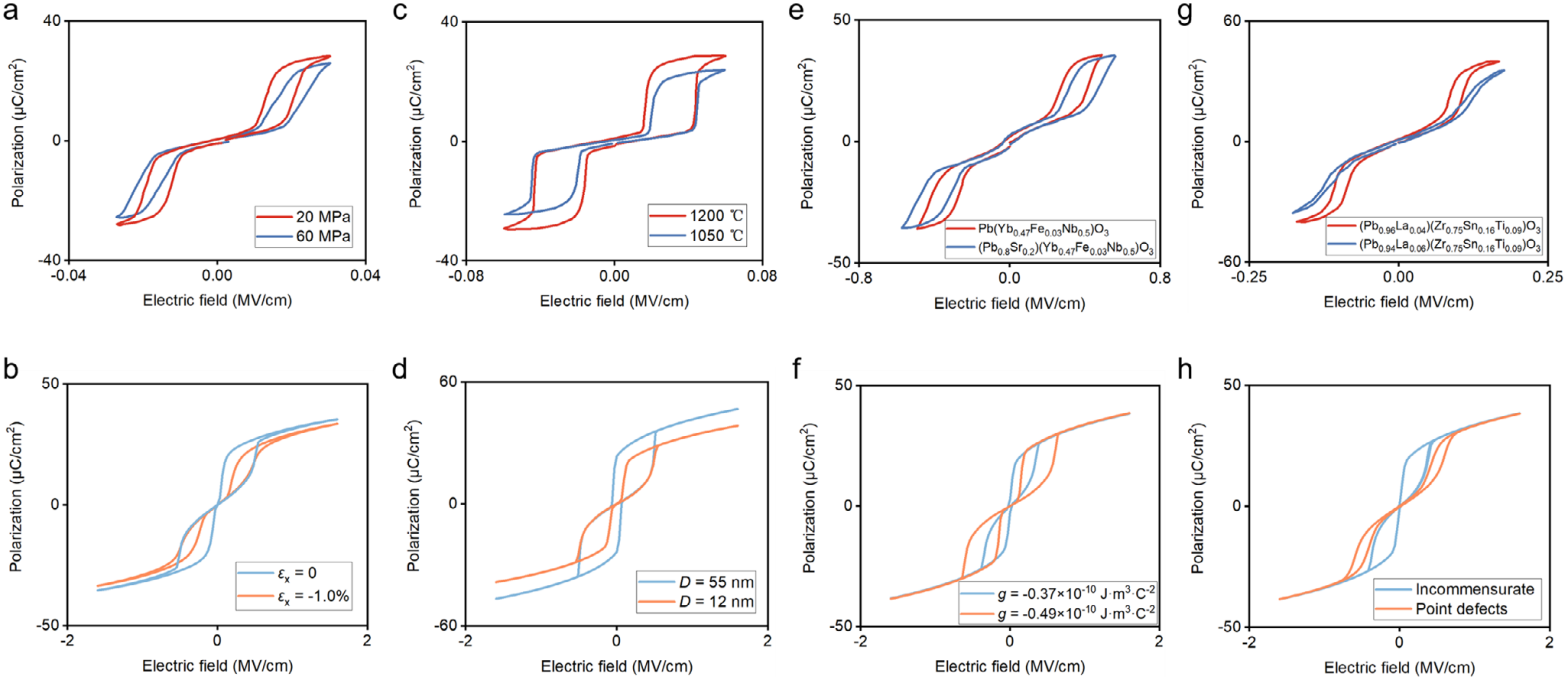
Comparison of *P*–*E* loops between phase‐field simulations and experimental results. (a) Effect of compressive stress applied along the external electric field direction on ceramics of (Pb_0.94‐x_La_0.04_Ba_x_)[(Zr_0.6_Sn_0.4_)_0.84_Ti_0.16_]O_3_ [19]_._ (b) Phase‐field simulations of uniaxial compressive strain applied along the external electric field direction. (c) Regulation of grain size in (Pb_0.97_La_0.02_)(Zr_0.65_Sn_0.25_Ti_0.10_)O_3_ ceramics by different sintering temperatures [54]. (d) Phase‐field simulations of grain size effect. (e) Improvement of energy storage performance in Sr‐doped Pb(Yb_0.47_Fe_0.03_Nb_0.5_)O_3_ ceramics [43]. (f) Phase‐field simulation of regulating antiferroelectric energy storage performance via gradient energy effect. (g) Improvement of energy storage performance in La‐doped Pb(Zr_0.75_Sn_0.16_Ti_0.09_)O_3_ ceramics [23]. (h) Phase‐field simulation of improving energy storage performance via point defect effect.

As discussed above, the multi‐scale regulation mechanism of transition points can be revealed through the *P*–*E* loops and domain structures obtained via phase‐field simulations. Similarly, relevant experimental results also provide evidence for the phase‐field simulations, as shown in Figure 5. In Figure 5a, a comparison of *P*–*E* loops of (Pb_0.94‐x_La_0.04_Ba_x_) [(Zr_0.6_Sn_0.4_)_0.84_Ti_0.16_] O_3_ ceramics under compressive stress applied along the electric field direction is presented. When the compressive stress increases from 20 to 60 MPa, the *P*_s_ decreases, the *E*_FA_ increases, and the reverse phase transition polarization decreases. The same trend is also observed in the phase‐field simulation results where uniaxial compressive strain is applied along the electric field direction (Figure 5b). In ceramic preparation experiments, low‐temperature sintering is generally used to inhibit grain growth. When the sintering temperature of (Pb_0.97_La_0.02_)(Zr_0.65_Sn_0.25_Ti_0.10_)O_3_ AFE ceramics decreases from 1200°C to 1050°C, the average polarization decreases significantly. The polarization at *TP*_FA_ decreases, while the transition electric field increases, respectively, as shown in Figure 5c,d. In addition, element doping is an effective method to improve energy storage performance in experiments, doping with specific elements can regulate *TP*_AF_ and *TP*_FA_ of AFEs. In Pb(Yb_0.47_Fe_0.03_Nb_0.5_)O_3_ ceramics, we have found that substituting Pb^2+^ with Sr^2+^ can shift both *TP*_AF_ and *TP*_FA_ toward the high electric field direction (Figure 5e), enhancing the stability of the AFE phase. The trend is close to the regulation of *P*–*E* loops by the antiphase domain boundary effect (Figure 5f), as Sr^2^ doping changes the phase transition energy barrier of AFE‐FE. The ionic radius of Sr^2+^(1.19 Å) is smaller than that of Pb^2+^(1.49 Å), which leads to the decrease of tolerance factor. The increase in the oxygen octahedral tilt angle leads to the stabilization of antiphase domain boundaries, while enhancing the *E*_AF_ and *E*_FA_. Substituting Pb^2+^ with La^3+^ is an effective way to enhance the antiferroelectricity of Pb‐based ceramics. Heterovalent La^3+^ can induce high density point defects in AFEs, significantly reducing hysteresis and achieving the synergistic improvement of *W*_rec_ and *η* (Figure 5g,h).

## 3 Conclusion

In this work, we have investigated the multi‐scale regulation strategy for the energy storage performance of PbZrO_3_‐based incommensurate AFE ceramics using a phase‐field‐machine learning model. We have realized the regulation of AFE (reverse) phase transition points through multiple factors, including local inhomogeneous strain, point defects, antiphase boundary energy, grain size, and external strain, thereby optimizing the *W*_rec_ and *η*. By training an energy storage performance prediction model and exploring the weight correlations among multiple factors via the XGBoost model, it is found that the biaxial compressive strain is the most critical regulatory variable, which can significantly reduce AFE hysteresis. Based on the phase‐field‐machine learning model, we have achieved the regulation of key phase transition parameter *E*_FA_ and revealed the domain dynamic mechanism underlying phase transition process regulation. Ultimately, under the comprehensive effect of multiple variables, an AFE ceramic with ultra‐high energy storage performance (*W*_rec_ of 22.1 J/cm^3^ and *η* of 96.1%) has been obtained. This study uncovers the phase transition dynamic mechanism of multi‐scale regulation methods for AFEs, establishes the intrinsic structure‐property correlation. It is expected to provide a theoretical basis for the experimental design of synergistic optimization of high energy storage density and efficiency.

## 4 Experimental Section

### 4.1 Phase‐Field Model

Phase‐field simulations were employed to investigate the electric field‐induced AFE‐FE phase transition process. Time‐dependent Ginzburg‐Landau (TDGL) equation was solved the for the temporal evolution of the polarization vector field:

(1) $$\frac{\partial P_{i}\left(r,t\right)}{\partial t}=-L\frac{\delta F}{\delta P_{i}\left(r,t\right)},\left(i=1,2,3\right)$$

where P_*i*_(*r*,*t*) was polarization, *L* was the kinetic coefficient, and *F* was the total free energy of the system, which was expressed as:

(2) $$F=\;\int \int \int \;\left(f_{\mathrm{Land}}+f_{\mathrm{elas}}+f_{\mathrm{grad}}+f_{\mathrm{elec}}\right)dV$$

where *V* was the system volume. The total energy functional comprised the Landau free energy density, gradient energy density, elastic energy density, and electrostatic energy density:

(3) $$f_{\mathrm{Land}}=\alpha_{i}P_{i}^{2}+\alpha_{\mathrm{ij}}P_{i}^{2}P_{j}^{2}+\alpha_{\mathrm{ijk}}P_{i}^{2}P_{j}^{2}P_{k}^{2}$$

where *P*_i_, *P*_j_ and *P*_k_ were polarization components. *α*_i_, *α*_ij_ and *α*_ijk_ were Landau coefficients.

The elastic energy density could be expressed as:

(4) $$f_{\mathrm{elas}}=\frac{1}{2}C_{\mathrm{ijkl}}\left(\varepsilon_{\mathrm{ij}}-\varepsilon_{\mathrm{ij}}^{0}\right)\left(\varepsilon_{\mathrm{kl}}-\varepsilon_{\mathrm{kl}}^{0}\right)$$

where *C*_ijkl_ was the elastic stiffness tensor, *ε* and *ε* ^0^ are the total local strain, and the eigenstrain, respectively. The eigenstrain was as follows:

(5) $$\varepsilon_{\mathrm{ij}}^{0}=Q_{\mathrm{ijkl}}P_{k}P_{l}+\varepsilon_{\mathrm{ij}}^{\mathrm{ex}}+\varepsilon_{\mathrm{ij}}^{\mathrm{rand}}$$

where *Q*_ijkl_ was the electrostrictive coefficient of the corresponding crystal. *ε* ^ex^ was the external strain applied, *ε* ^rand^ was the local inhomogeneous strain induced by elements doping [35]. The *ε* ^rand^ followed a Gaussian distribution. To solve the equilibrium heterogeneous strain field *δε*_ij_, a set of displacements *u*_i_(*x*) had been introduced,

(6) $$\delta \varepsilon_{\mathrm{ij}}=\frac{1}{2}\left(u_{i,j}+u_{j,i}\right)$$

the mechanical equilibrium condition was given by:

(7) $$\sigma_{\mathrm{ij},j}=0$$

where *σ*_ij_ was the elastic stress. The gradient energy density could be expressed as [36]:

(8) $$\begin{array}{c} f_{\mathrm{grad}}=\beta_{11}\sum_{i}P_{i}^{2}P_{i,i}^{2}+\beta_{12}\sum_{i\neq j\neq k}P_{i}^{2}\left(P_{j,k}^{2}+P_{k,j}^{2}\right)+\gamma_{11}\sum_{i}\theta_{i}^{2}P_{i,i}^{2}+\gamma_{12}\sum_{i\neq j\neq k}\theta_{i}^{2}\left(P_{j,k}^{2}+P_{k,j}^{2}\right)\;+\varphi_{11}\sum_{i}P_{i,\mathrm{ii}}^{2}+\varphi_{12}\sum_{i\neq j}P_{i,\mathrm{jj}}^{2} \end{array}$$

*θ*_i_ was the oxygen tilt. *β*_11_ and *β*_12_ were the positive constants, which described polar‐polar interactions. *γ*_11_ and *γ*_12_ are the negative constants, which described the coupling between oxygen tilt and polarizations. *φ*_11_ and *φ*_12_ were the positive constants of high‐order gradient energy describing next‐nearest‐neighbor interaction between polarizations, which could drive the phase transition from AFE to FE. For the convergence of the numerical solution, we use Equation 9 to normalize the gradient energy coefficients,

(9) $$\lambda =\frac{\gamma \cdot \theta^{2}}{{\left(a_{c}\right)}^{2}\cdot a_{0}},\;\zeta =\frac{\varphi }{{\left(a_{c}\right)}^{4}\cdot a_{0}}$$

where 5.54 × 10^7^ J·m·C^−2^ for *a*_0_. *a*_c_ equals to 0.5 nm. *λ* and *ζ* were dimensionless gradient energy coefficients. We could conclude that the first‐order of the gradient energy was always smaller than zero in AFE materials. To simplify the model, we assume that *β*_11_ = *β*_12_ = 0. Thus, Equation 8 could be simplified as follows.

(10) $$\begin{array}{c} f_{\mathrm{grad}}=\gamma_{11}\sum_{i}\theta_{i}^{2}P_{i,i}^{2}+\gamma_{12}\sum_{i\neq j\neq k}\theta_{i}^{2}\left(P_{j,k}^{2}+P_{k,j}^{2}\right)+\varphi_{11}\sum_{i}P_{i,\mathrm{ii}}^{2}+\varphi_{12}\sum_{i\neq j}P_{i,\mathrm{jj}}^{2} \end{array}$$

To unify the expression of coupling coefficient between oxygen tilt and polarizations, we used *g* to denote *γ*·*θ* ^2^. To investigate PbZrO_3_‐based incommensurate AFEs, we choose *φ* equals to 3.46 × 10^−30^ J·m^5^·C^−2^ in the subsequent simulations.

The electrostatic energy density was given by:

(11) $$f_{\mathrm{elec}}=-\frac{1}{2}P_{i}E_{i}^{\mathrm{in}}-P_{i}E_{i}^{\mathrm{ext}}-P_{i}E_{i}^{\mathrm{rand}}$$

where *E* ^in^ was the dipole‐dipole interaction electric field, *E* ^ext^ was the external electric field, *E* ^rand^ was the local electric field caused by the random point defects [37, 38, 39]. The construction form of the local electric field generated by point defects in the phase‐field model can be found in Figure S9. The electric field strength followed a Gaussian distribution, and the electric field direction is random. In this work, we used the standard deviation *c* of the Gaussian distribution to describe the point defect concentration.

The equation was solved by a semi‐implicit Fourier spectral method. We adopt the periodic boundary condition in both the X and Y directions. We use Landau coefficients of the Pb(Zr_1‐x_Ti_x_)O_3_ (x ≤ 0.1) system modified for the calculation [40]. *α*_1_ = −5.54 × 10^7^ J·m·C^−2^, *α*_11_ = 5.60 × 10^8^ J·m^5^·C^−4^, *α*_12_ = 2.89 × 10^8^ J·m^5^·C^−4^, *α*_111_ = 1.65 × 10^9^ J·m^9^·C^−6^, *α*_112_ = −8.66 × 10^8^ J·m^9^·C^−6^, *α*_123_ = 3.19 × 10^10^ J·m^9^·C^−6^, *C*_11_ = 15.6 × 10^10^ N/m^2^, *C*_12_ = 9.6 × 10^10^ N/m^2^, *C*_44_ = 12.7 × 10^10^ N/m^2^, *Q*_11_ = 0.048 m^4^/C^2^, *Q*_12_ = −0.015 m^4^/C^2^, *Q*_44_ = 0.047 m^4^/C^2^.

### 4.2 Machine Learning Model

Before model training, the data had been separated into features and target variables. A stratified sampling method was used to split the data into training (70%) and testing sets (30%). XGBoost algorithm had been adopted as the prediction model. Based on the gradient boosting framework, this model achieved efficient regression prediction by constructing an ensemble of multiple decision trees, and was particularly suitable for handling nonlinear relationships and high‐dimensional feature data. To optimize model performance, GridSearchCV had been employed for hyperparameter tuning. The specific parameter search space was set as follows. The number of trees: 100, 200, and 300. The maximum depth: 4, 5, and 6. The learning rate: 0.05, 0.1, and 0.2. In this work, three gradient boosting tree models had been compared, all of which belong to the ensemble learning framework. These models improved predictive performance by constructing an ensemble of multiple decision trees, specifically including XGBoost, LightGBM, and CatBoost. In the comparative data, the parameter settings were consistent across all models: the number of trees is100, the maximum depth is 3, and the learning rate is 0.1.

## Author Contributions

K. Xu and H. Huang contributed to the design of this study, the acquisition and interpretation of the supporting data, and the drafting of the text. X. Shi, Z. Xi, C. Guo, R. Gao, L. Yang, J. Wang and D. Zhou contributed to the writing and data interpretation.

## Conflicts of Interest

The authors declare no conflict of interest.

## Supporting information




**Supporting File**: advs74314‐sup‐0001‐SuppMat.docx.

## Data Availability

The data that support the findings of this study are available from the corresponding author upon reasonable request.
