# Supplementary material for: Machine‐Learning‐Guided Design of Incommensurate Antiferroelectrics via Field‐Driven Phase Engineering
Source: Adv Sci (Weinh). 2026 Feb 9;13(22):e23873. doi: 10.1002/advs.202523873 (PMC13088312; doi:10.1002/advs.202523873)
Supplement: Supplementary file 1 — Supporting File: advs74314‐sup‐0001‐SuppMat.docx. [file ADVS-13-e23873-s001.docx]

**Supplementary Information**

Ke Xu ^1^, Xiaoming Shi ^2^, Zhaochen Xi ^3^, Shouzhe Dong ^1^, Changqing Guo ^1^, Rongzhen Gao ^1^,

Letao Yang ^1^, Jing Wang ^1^, Di Zhou^3^, and Houbing Huang ^1^*

^1^ School of Materials Science and Engineering & School of Interdisciplinary Science, Beijing Institute of Technology, Beijing 100081, China.

^2^ School of Mathematics and Physics, University of Science and Technology Beijing, Beijing 100083, China.

^3^ School of Electronic Science and Engineering, Xi’an Jiaotong University, Xi’an 710049, China.

*Corresponding author email: [hbhuang@bit.edu.cn](mailto:hbhuang@bit.edu.cn)

**Supplementary Figures**


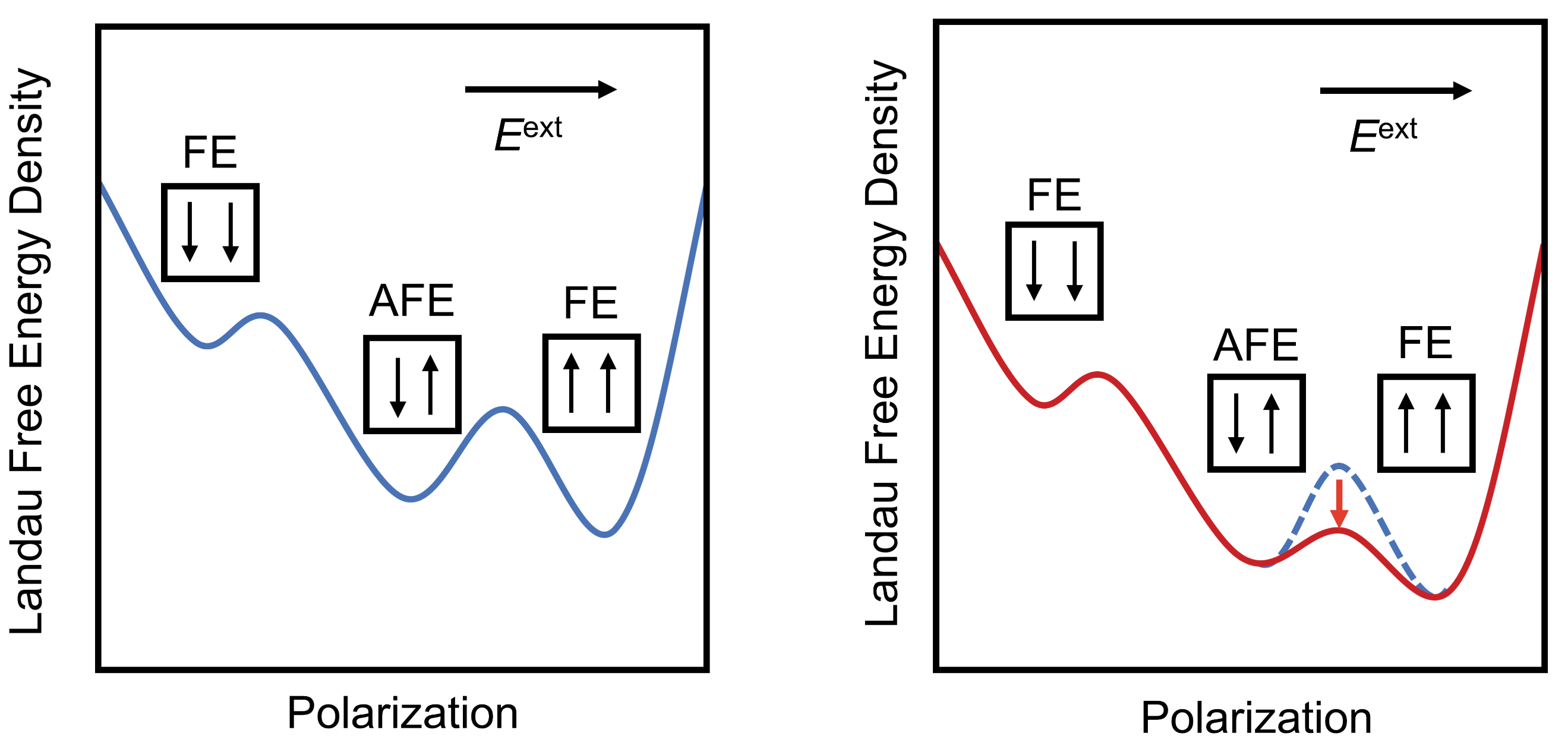


**Figure S1. Schematic illustrating the mechanism of improved energy storage performance revealed by the Landau free energy density.**


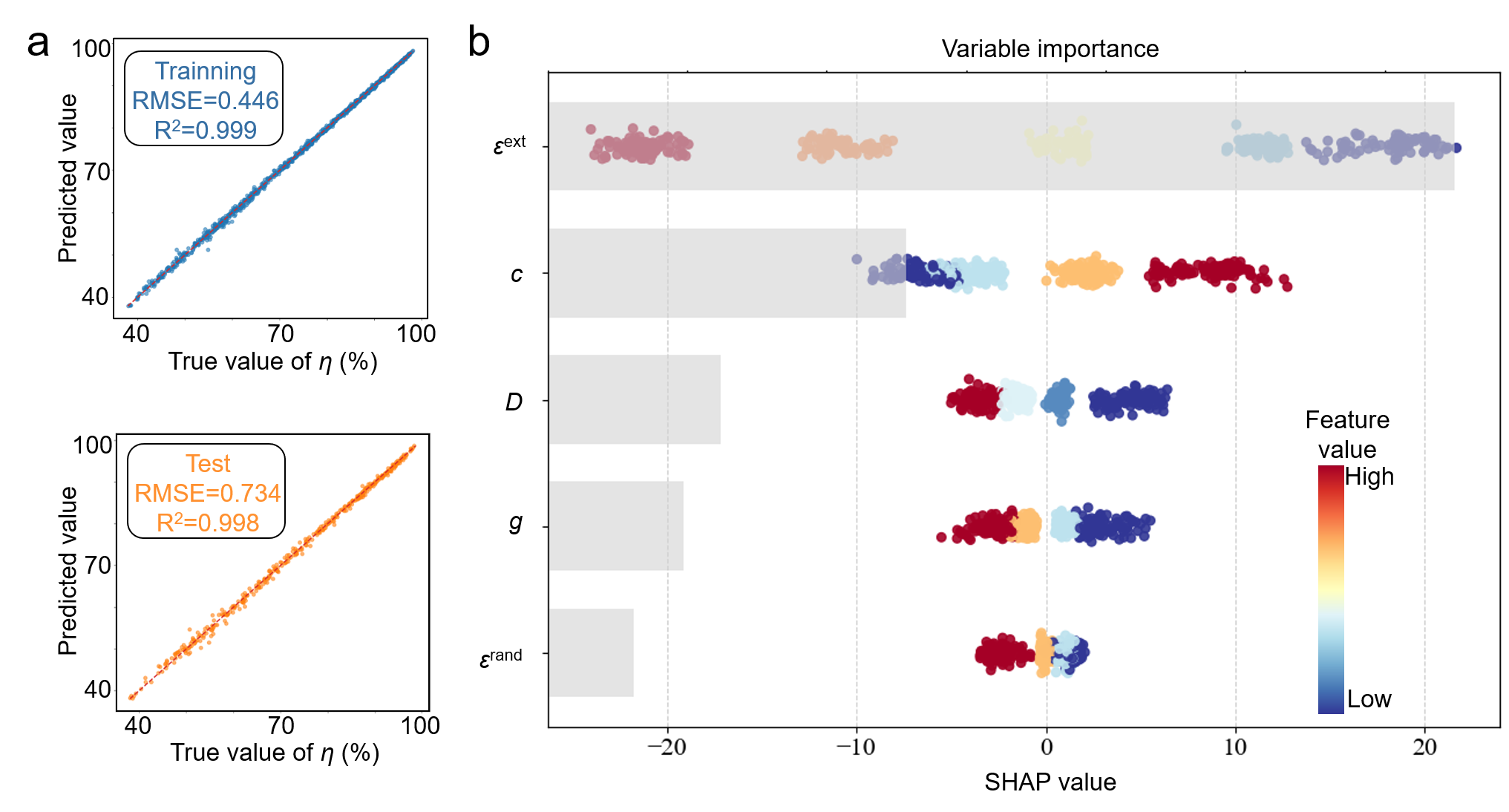


**Figure S2. XGBoost regression model for decoupling the key factors determining energy storage efficiency.** **a** Comparison of *η* between the phase-field simulations and XGBoost prediction model. **b** SHAP summary plot for the XGBoost model of *W*_rec_ including variable importance and SHAP value.


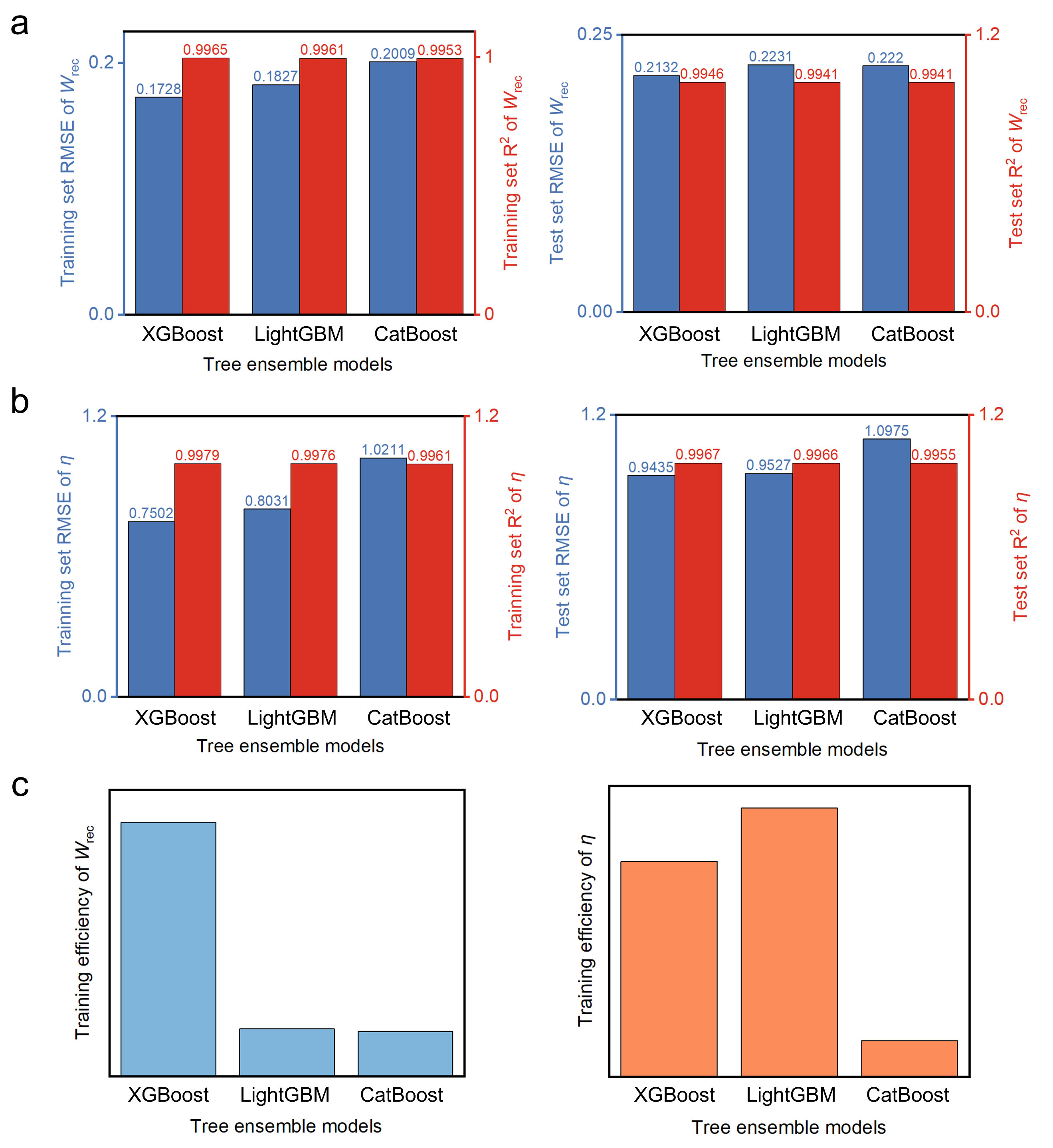


**Figure S3. Comparison of tree ensemble models (XGBoost, LightGBM and CatBoost) in AFEs energy storage performance.** **a** Comparison of RMSE and R^2^ for *W*rec. **b** Comparison of RMSE and R^2^ for *η*. **c** Comparison of training efficiency.

**
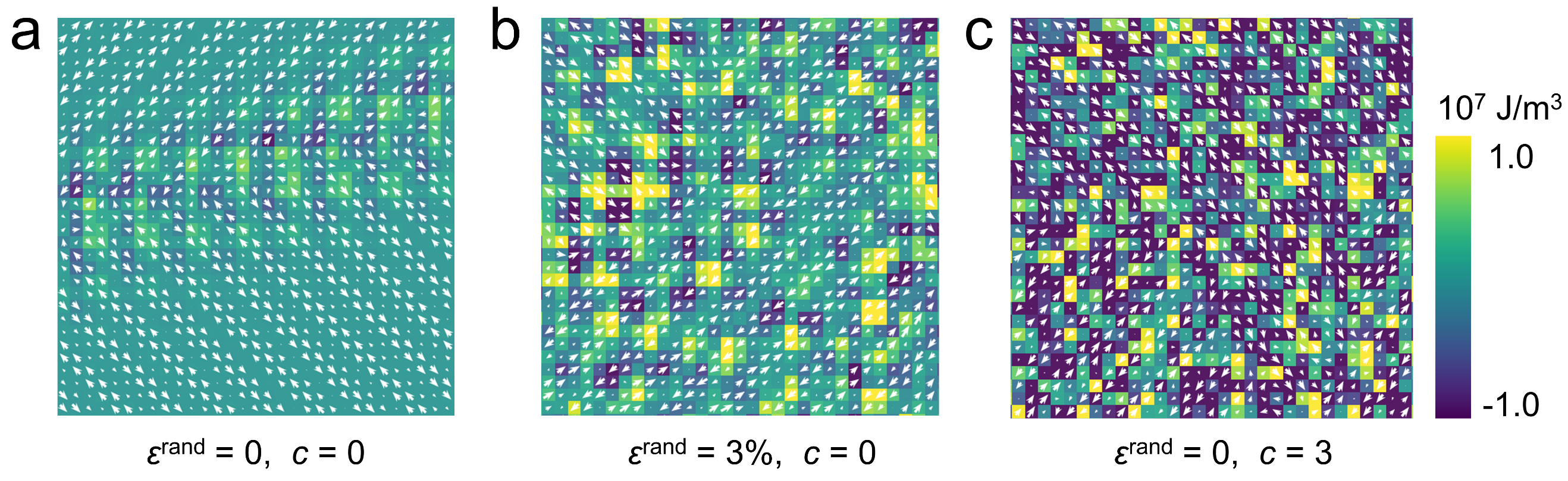
**

**Figure S4. Electrostatic energy density distribution under dipole-scale factors a** domain structure of control group, **b** inhomogeneous strain (*ε*^rand^ = 3%) and **c** point defect concentration (*c* = 3).

**
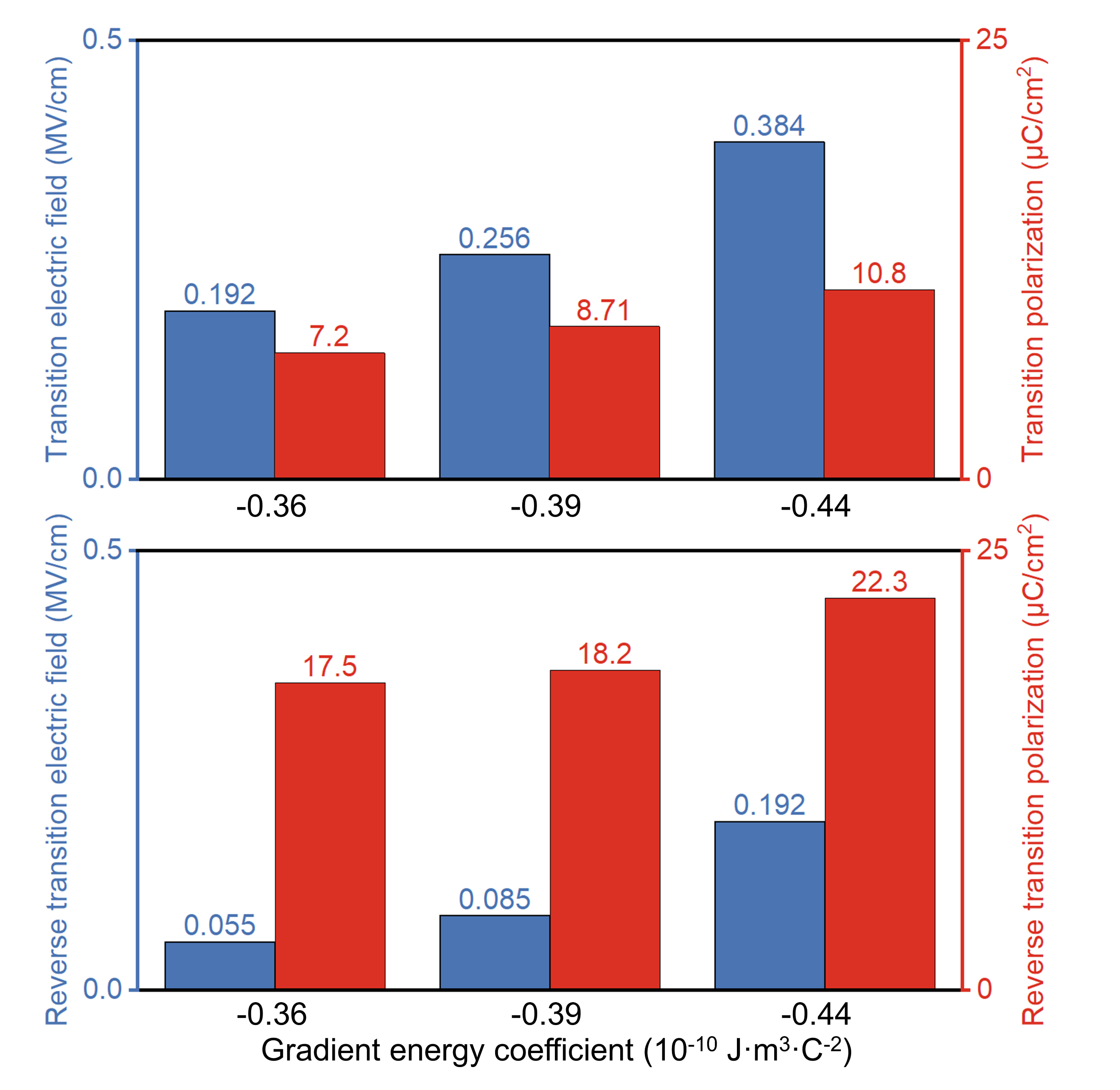
**

**Figure S5. Statistical chart of *TP*_AF_ and *TP*_FA_ for energy storage performance regulation by different gradient energy coefficients.**

**
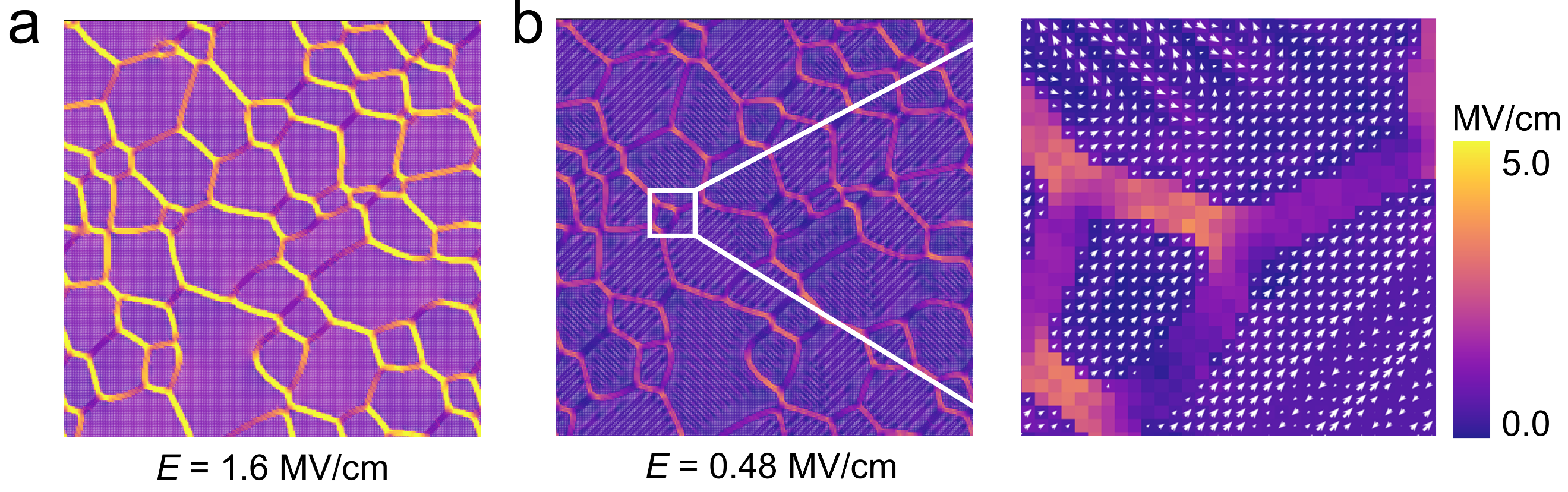
**

**Figure S6. Electric field strength and polarization distributions under the external electric field applied.** **a** *E* = 1.6 MV/cm. **b** *E* = 0.48 MV/cm.

**
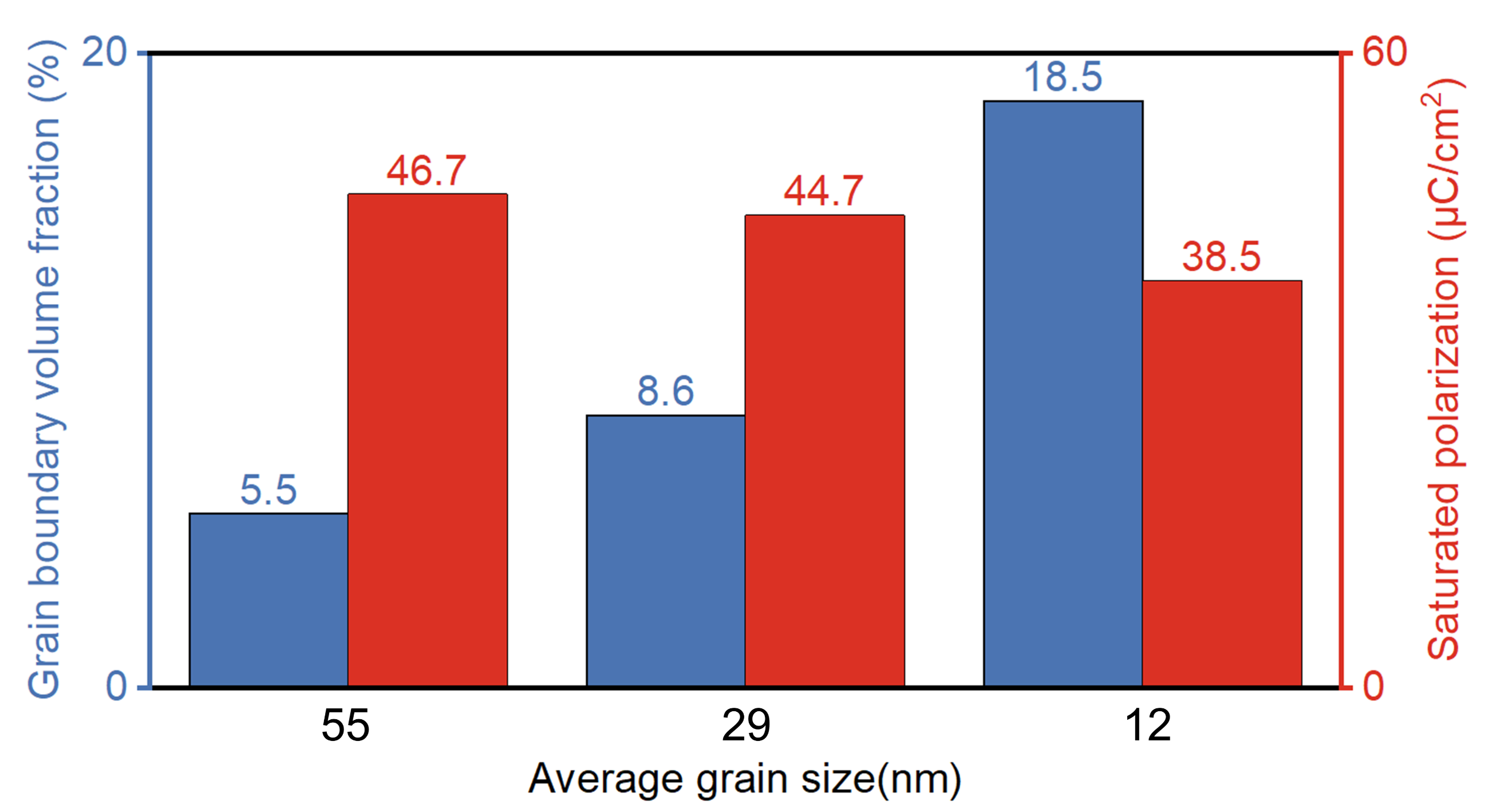
**

**Figure S7. Statistical chart of grain boundary volume fraction and *P*_s_ with different average grain size.**

**
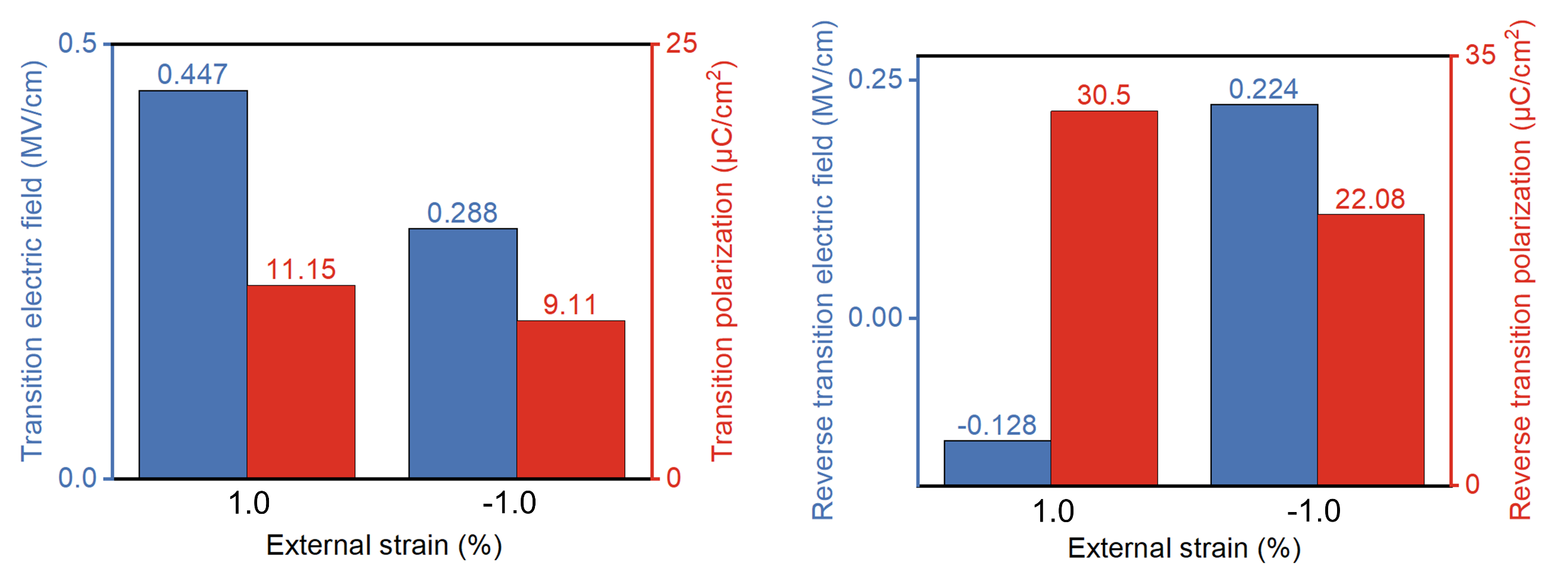
**

**Figure S8. Statistical chart of *TP*_AF_ and *TP*_FA_ for energy storage performance regulation by external strain manipulation.**

The probability density function is expressed as follows,

$$f\left(E^{*}\right)=\frac{1}{\sqrt{2\pi c^{2}}}\exp\left(-\frac{\left(E^{*}-\mu\right)^{2}}{2c^{2}}\right)$$

*f* denotes the probability density function, and *E** represents the dimensionless electric field strength. *μ* is the mean value. Typically, the random point field exhibits no macroscopic bias, and thus *μ* = 0. A larger standard deviation *c* corresponds to a higher probability of deviating from electroneutrality and a greater concentration of point defects.


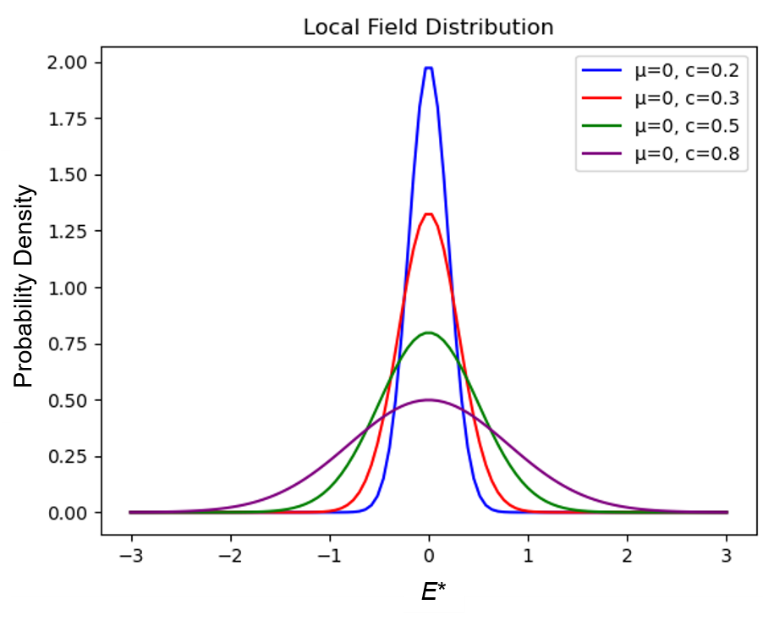


**Figure S9. Schematic diagram of Gaussian probability density distribution for local random electric field.**
